# Mieap-induced accumulation of lysosomes within mitochondria (MALM) regulates gastric cancer cell invasion under hypoxia by suppressing reactive oxygen species accumulation

**DOI:** 10.1038/s41598-019-39563-x

**Published:** 2019-02-26

**Authors:** Keiichiro Okuyama, Yoshihiko Kitajima, Noriyuki Egawa, Hiroshi Kitagawa, Kotaro Ito, Shinichi Aishima, Kazuyoshi Yanagihara, Tomokazu Tanaka, Hirokazu Noshiro

**Affiliations:** 1 Department of Surgery, Saga University Faculty of Medicine, 5-1-1 Nabeshima, Saga, 849-8501 Japan; 2 Department of Surgery, National Hospital Organization Higashisaga Hospital, 7324 Harakoga, Miyaki-cho, Miyaki-gun, Saga, 849-0101 Japan; 3 Department of Pathology, Saga University Faculty of Medicine, 5-1-1 Nabeshima, Saga, 849-8501 Japan; 4 Division of Translational Research, Exploratory Oncology Research & Clinical Trial Center, National Cancer Center, 6-5-1 Kashiwanoha, Kashiwa, Chiba 277-8577 Japan

## Abstract

Mitochondrial quality control (MQC) protects against potentially damaging events, such as excessive generation of mitochondrial reactive oxygen species (mtROS). We investigated the contribution of the two major MQC processes, namely, mitophagy and Mieap-induced accumulation of lysosomes within mitochondria (MALM), to the response to hypoxia of two human gastric cancer (GC) cell lines. We found that hypoxia increased mtROS generation and cell invasion in 58As9, but not in MKN45, although the transcription factor hypoxia-inducible factor 1α was induced in both cell lines. Colocalisation of lysosomes with mitochondria was found only in hypoxic MKN45 cells, suggesting that hypoxia-induced MQC functions normally in MKN45 but may be impaired in 58As9. Hypoxia did not lead to decreased mitochondrial mass or DNA or altered appearance of autophagosomes, as judged by electron microscopy, suggesting that mitophagy was not induced in either cell line. However, western blot analysis revealed the presence of the MALM-associated proteins Mieap, BNIP3 and BNIP3L, and the lysosomal protein cathepsin D in the mitochondrial fraction of MKN45 cells under hypoxia. Finally, Mieap knockdown in MKN45 cells resulted in increased mtROS accumulation and cell invasion under hypoxia. Our results suggest that hypoxia-induced MALM suppresses GC cell invasion by preventing mtROS generation.

## Introduction

Mitochondria play crucial roles in maintaining cellular homeostasis by regulating diverse processes such as energy production, cell signalling and apoptosis^1,2^. These organelles are also a major source of intracellular reactive oxygen species (ROS), which include highly reactive free oxygen radicals, such as the superoxide anion (O_2_·^−^) and the hydroxyl radical (OH·), as well as stable nonradical oxidants such as hydrogen peroxide (H_2_O_2_)^3,4^. ROS are commonly produced as by-products of oxidative phosphorylation^1,2^, but excessive ROS generation in the mitochondria (mtROS) can lead to oxidative damage to proteins, lipids and DNA, sometimes resulting in apoptosis^1,2^. In addition, ROS accumulation is known to contribute to various diseases, such as degenerative disorders and cancer^2,5^. Recent reports suggest that elevated levels of mtROS promote cancer cell invasion and metastasis via the activation of several major signalling pathways and transcription factors^6–8^.

Hypoxia is a common characteristic of the microenvironment of solid tumours and leads to increased generation of mtROS by cancer cells^9,10^. In response to hypoxia, levels of the transcription factor hypoxia-inducible factor (HIF)-1 increase, leading to the transcription of genes that regulate oxygen homeostasis and promote the survival of cancer cells^11–16^. HIF-1 is a heterodimer composed of a constitutively expressed HIF-1β subunit and O_2_-regulated HIF-1α. Under normoxic conditions, HIF-1α is maintained at low levels via hydroxylation by the O_2_ sensor prolyl hydroxylase 2 (PHD2), which triggers its degradation via the ubiquitin–proteasome pathway^11,12,16^. Under hypoxic conditions, however, the low O_2_ tension inactivates PHD2 and HIF-1α is thus stabilised^11,12^. Elevation of mtROS also stabilises HIF-1α since PHD2 is inactivated by the oxidation of Fe(II) in its catalytic centre^17–19^. Thus, mtROS regulation of HIF-1α is a pivotal mechanism underlying cancer progression under hypoxia^19^. Indeed, a notable study by Ishikawa *et al*.^20^ demonstrated the importance of mtROS in tumour cell metastasis in mouse. The authors replaced endogenous mitochondria from a poorly metastatic tumour cell line with those from a highly metastatic cell using cybrid technology^20^. Impairment of the electron transport chain by mutation of mtDNA genes in these cells induced excessive ROS production and increased metastatic ability^20^, suggesting that mitochondrial integrity and activity are important determinants of the metastatic ability of cancer cells.

Mitochondrial quality control (MQC) pathways are essential for monitoring and maintaining mitochondrial integrity under conditions of stress, in part by preventing excessive mtROS production^21^. Mitophagy, an organelle-specific form of autophagy, is a major mechanism of MQC activated in response to certain physiological stresses, such as hypoxia and nutrient deprivation^22–24^_._ During mitophagy, the entire mitochondria are engulfed in double-membraned autophagosomes and ultimately degraded in lysosomes^22–24^. Recent studies demonstrated that hypoxia-induced mitophagy requires HIF-1α-dependent expression of BNIP3, a BCL2 family member^25,26^. Importantly, defects in mitophagy arising from the loss of BNIP3 are known to promote metastasis in mammary tumours^27^. In addition, Liu *et al*.^28^ reported that the mitochondrial membrane protein FUNDC1 plays a critical role in hypoxia-induced mitophagy through a mechanism distinct from that played by BNIP3^28^.

Miyamoto *et al*.^29^ described another MQC mechanism in which the p53 target gene mitochondria-eating protein (Mieap) induced the accumulation of lysosomal proteins within the mitochondria of colorectal cancer cell lines in response to γ-irradiation, resulting in the elimination of oxidised mitochondrial proteins, repair of the unhealthy mitochondria and improvement of mitochondrial respiration^29,30^. In this process, termed MALM (Mieap-induced accumulation of lysosomes within mitochondria), interaction of Mieap with BNIP3 or BNIP3L facilitates the translocation of lysosomal proteins into the mitochondria by inducing pore formation in the mitochondrial double membrane^30–32^. However, it is not known whether MALM is induced in cancer cells by hypoxia or whether loss of MALM accelerates cancer progression under hypoxic conditions. We previously reported that impairment of hypoxia-induced MQC is positively correlated with hypoxia-induced invasion in gastric cancer (GC) cells. However, we did not investigate the MQC mechanism underlying this^33^.

In the present study, we sought to identify the mechanism of hypoxia-induced MQC using two GC cell lines (58As9 and MKN45) that differ in their ability to invade, an initial step towards cancer metastasis. We investigated the effects of hypoxia on mtROS generation and invasion and determined the contribution of mitophagy and MALM to hypoxia-induced MQC in these cells.

## Results

### Effects of hypoxia on invasion and ROS generation by GC cell lines

**Figure 1:**
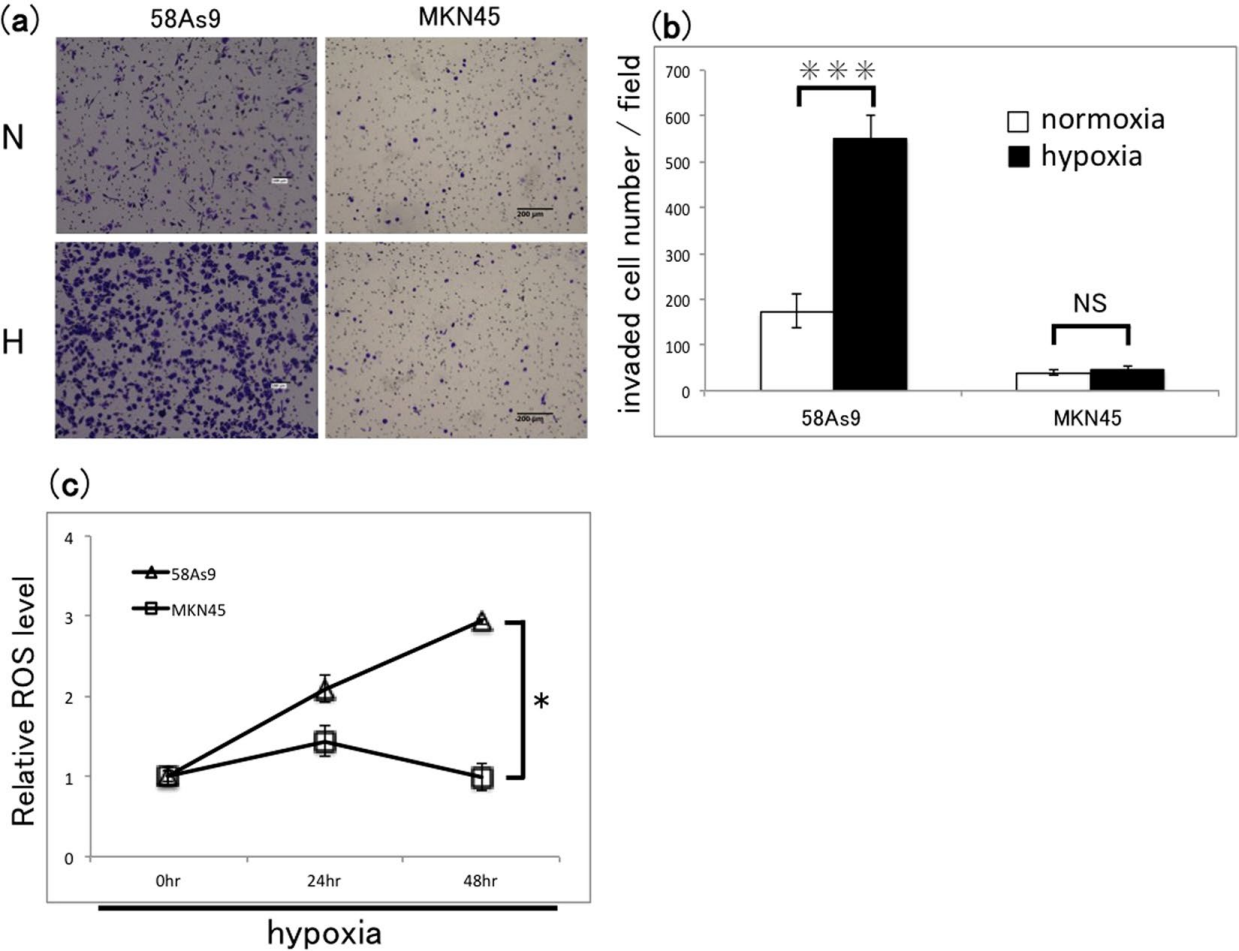
Analysis of hypoxia-induced cell invasion and ROS accumulation in GC cell lines. (**a**) Transwell invasion assay of 58As9 and MKN45 GC cells after incubation under normoxia (N) or hypoxia (H) for 48 h. Cells were stained with crystal violet. Scale bars, 200 µm. (**b**) Quantification of invaded cells shown in (**a**). Mean ± SD of n = 3. ***P < 0.005; NS, not significant. (**c**) Flow cytometric analysis of total intracellular ROS in 58As9 and MKN45 cells after incubation under normoxia (0 h) and hypoxia for 24 and 48 h. Data are normalised to the levels under normoxia. Mean ± SD of n = 3. *P < 0.05.

To determine how hypoxia affects the invasiveness of the two GC cell lines 58As9 and MNK45, we performed Transwell invasion assays by incubating cells for 48 h under conditions of normoxia (5% CO_2_ in air) or hypoxia (1% O_2_ and 5% CO_2_ in N_2_). As shown in Fig. 1, hypoxia significantly increased the number of invaded 58As9 cells, whereas MKN45 cells were unaffected (Fig. 1a,b). Measurement of total intracellular ROS levels using a 2′,7′-dichlorofluorescein diacetate-based flow cytometric assay indicated that exposure to hypoxia for up to 48 h significantly increased ROS production in 58As9 cells, while ROS levels in MKN45 cells were only marginally increased (Fig. 1c). These findings indicate that 58As9 cells respond to hypoxia by increasing mtROS production and cell invasion, whereas MKN45 cells are relatively unaffected by hypoxia.

### mtROS generation and MQC status in GC cells exposed to hypoxia

**Figure 2:**
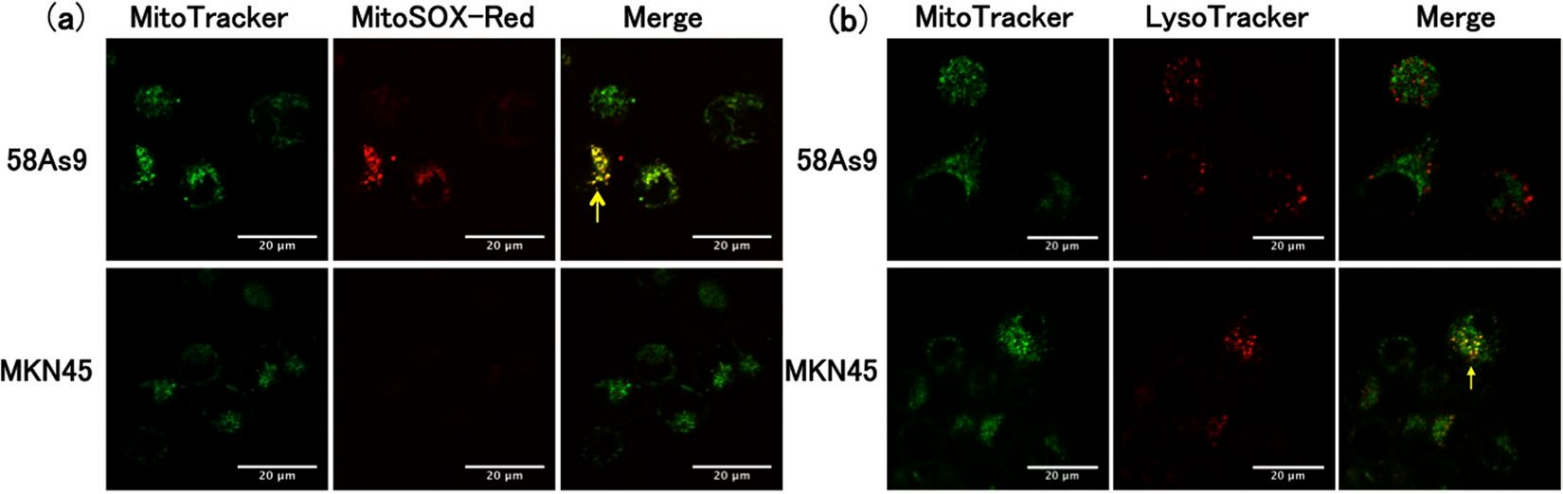
mtROS production and MQC status in GC cells. (**a**) Fluorescence micrographs of 58As9 and MKN45 cells after incubation under hypoxia for 48 h and double staining with MitoTracker and MitoSOX-Red. Colocalisation of fluorescence, reflecting mitochondrial ROS production, is indicated by the arrow in the merged image of 58As9 cells. Scale bars, 20 µm. (**b**) Fluorescence images of 58As9 and MKN45 cells after incubation under hypoxia for 48 h and double staining with MitoTracker and LysoTracker. Dotted colocalisation of fluorescence, reflecting the presence of mitochondrial lysosomes, is indicated by the arrow in the merged image of MKN45 cells. Scale bars, 20 µm.

To determine whether the increase in total intracellular ROS resulted from mtROS generation, we labelled the cells with fluorescent markers of mitochondria (MitoTracker) and mtROS (MitoSOX Red) and examined their colocalisation by microscopy. Consistent with the results of the flow cytometric assay, we observed the colocalisation of MitoSOX Red and MitoTracker fluorescence in several 58As9 cells, but not MKN45 cells, after incubation for 48 h under hypoxia (Fig. 2a). MitoSOX Red fluorescence was not increased in either cell line incubated under normoxia (data not shown).

We next investigated whether the difference in responses to hypoxia between the two cell types might be due to differences in their MQC activity. To this end, cells were exposed to hypoxia for 48 h and the colocalisation of mitochondria and lysosomes was assessed by double staining with MitoTracker and LysoTracker, a fluorescent lysosome marker. Notably, dotted colocalisation of the fluorescent signals was observed in several MKN45 cells, but not in 58As9 cells (Fig. 2b). Under normoxia, neither cell line showed overlapping MitoTracker and LysoTracker signals (data not shown). Taken together, these data indicate that hypoxia induces mtROS generation in 58As9 cells but not in MKN45 cells, whereas it induces the formation of mitochondrial lysosomes in MKN45 cells but not in 58As9 cells; thus, MKN45 cells appear to mount a more vigorous MQC response than 58As9 cells.

### Effect of N-acetyl-l-cysteine (NAC) treatment on mtROS generation and invasion in 58As9 cells

**Figure 3:**
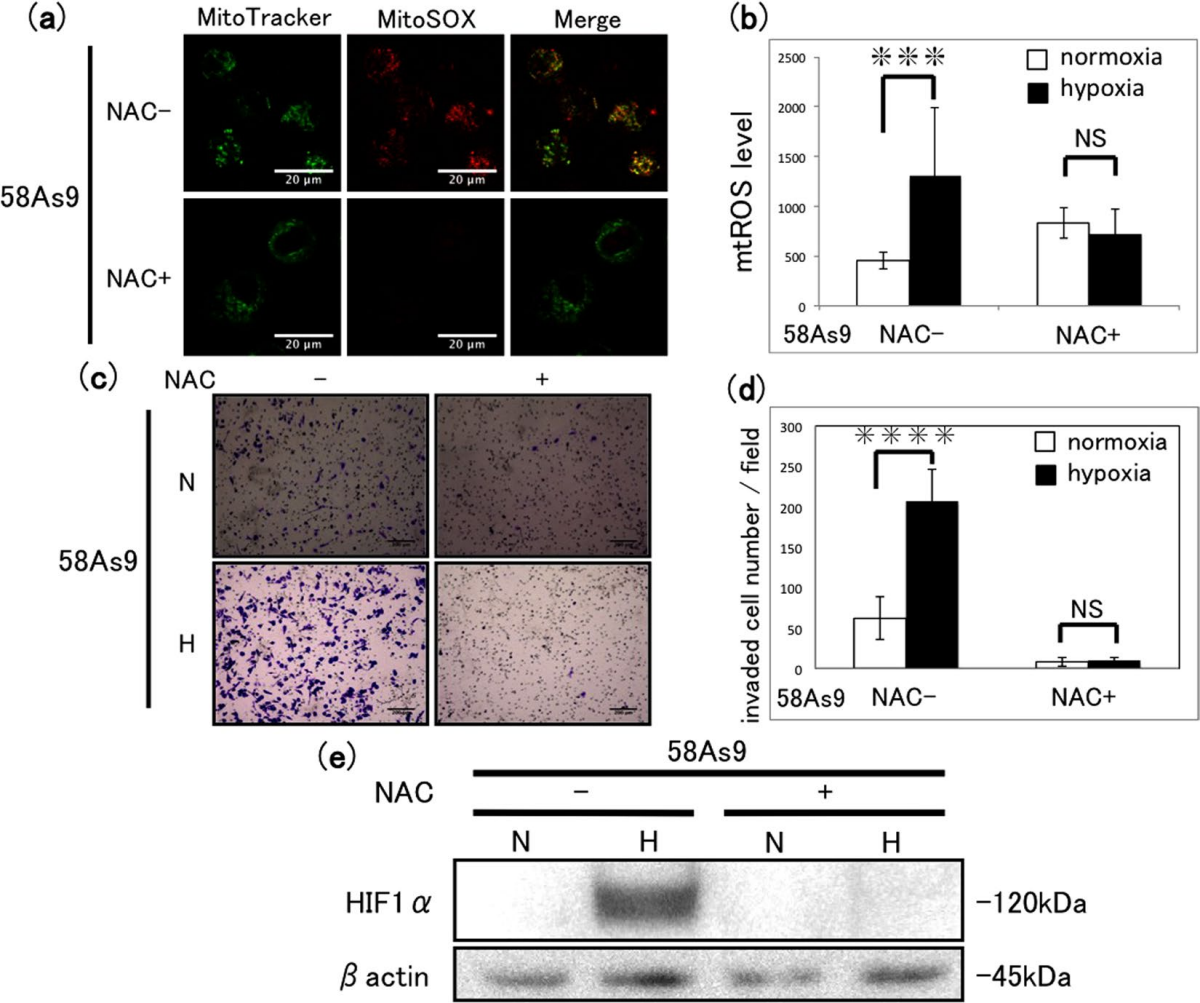
Effect of a ROS scavenger on mtROS generation and invasion in 58As9 cells. (**a**) Fluorescence micrographs of 58As9 cells after incubation with (+) or without (−) 20 mM N-acetyl-l-cysteine (NAC) for 48 h and double staining with MitoTracker and MitoSOX-Red. Scale bars, 20 µm. (**b**) Quantification of mtROS levels in untreated and NAC-treated cells after incubation under normoxia (N) or hypoxia (H) for 48 h. Mean ± SD of n = 3. NS, not significant; ***P < 0.005. (**c**) Transwell invasion assay of 58As9 cells after incubation with or without 20 mM NAC under normoxia (N) or hypoxia (H) for 48 h. Scale bars, 200 µm. (**d**) Quantification of invaded cells shown in (**c**). Mean ± SD of n = 3. NS, not significant; ****P < 0.001. (**e**) Western blot analysis of HIF-1α expression after incubation of cells with (+) or without (−) 20 mM NAC under normoxia (N) or hypoxia (H) for 12 h.

To determine whether the production of mtROS by 58As9 cells directly regulates hypoxia-induced invasion, we treated the cells with NAC, a ROS scavenger, and measured invasion under normoxic and hypoxic conditions. First, we confirmed that NAC effectively reduced mtROS generation using the fluorescence assay. Indeed, while exposure to hypoxia in the absence of NAC significantly increased mtROS generation, the response was inhibited by NAC treatment (Fig. 3a,b). Interestingly, NAC had the same effect on 58As9 cell invasion measured after incubation for 48 h under hypoxia or normoxia (Fig. 3c,d), suggesting that elevated mtROS levels were required for invasion. We also observed that cell proliferation was significantly decreased in NAC-treated 58As9 cells compared with that in control, untreated cells; however, this was not accompanied by an increase in cell death (Supplementary Fig. S1). In addition, we assessed the effects of NAC on HIF-1α protein levels by western blot analysis. Exposure to hypoxia for 48 h induced HIF-1α expression in untreated 58As9 cells, but this was strongly inhibited by NAC treatment (Fig. 3e). Collectively, these findings suggest that the inhibition of mtROS generation by NAC treatment reduces cell invasion, cell proliferation and HIF-1α expression in 58As9 cells under hypoxia.

### Effect of the lysosomal inhibitor chloroquine (CQ) on hypoxia-induced MQC in MKN45 cells

**Figure 4:**
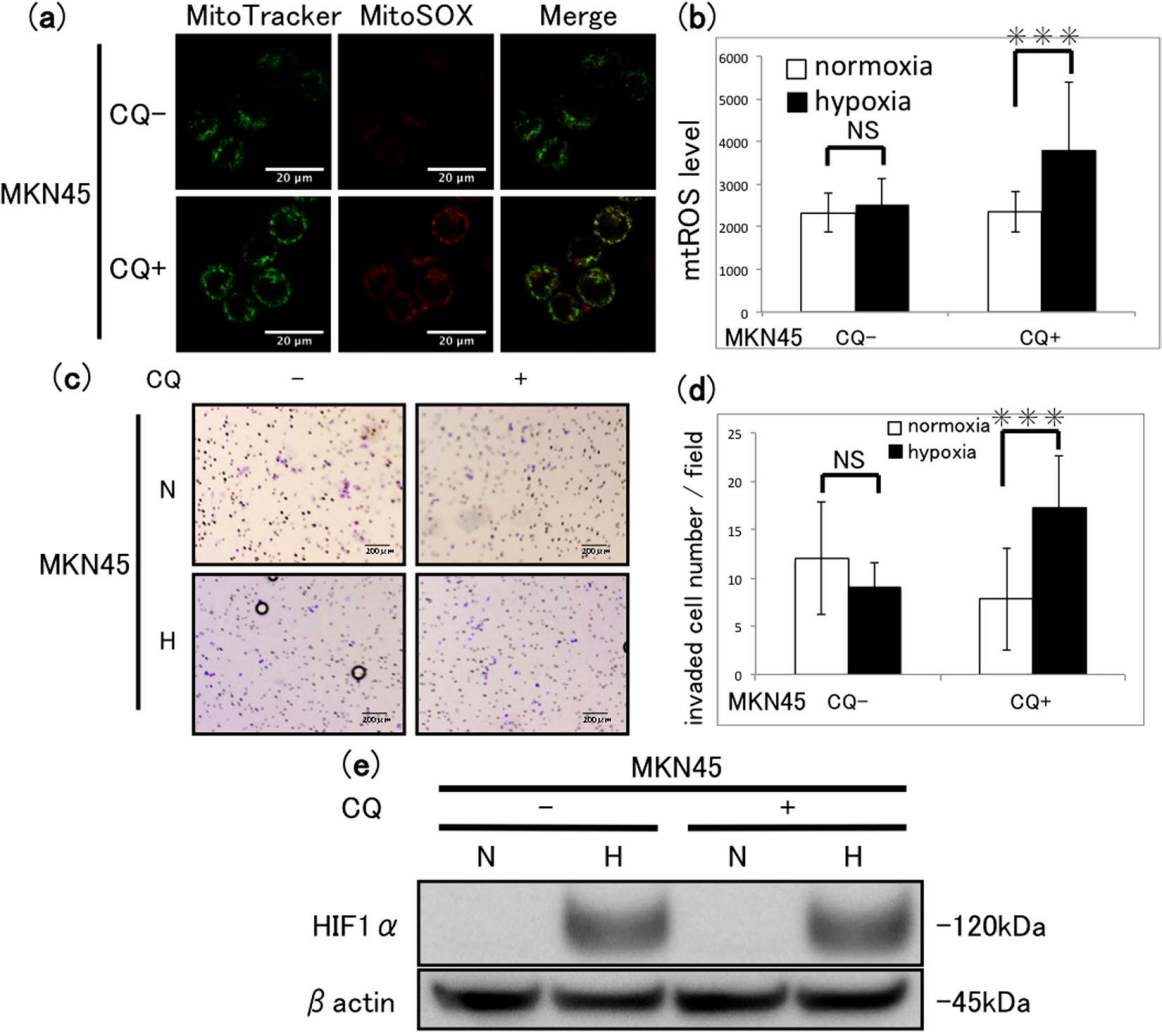
Effect of lysosomal inhibition on mtROS generation and invasion of MKN45 cells. (**a**) Fluorescence micrographs of cells after incubation with (+) or without (−) 10 µM chloroquine (CQ) for 48 h of hypoxia and double staining with MitoTracker and MitoSOX-Red. Scale bars, 20 µm. (**b**) Quantification of mtROS levels in cells treated with or without 10 µM CQ under normoxia (N) or hypoxia (H) for 48 h. Mean ± SD of n = 3. NS, not significant; ***P < 0.005. (**c**) Transwell invasion assay of MKN45 cells after incubation with or without 10 µM CQ under normoxia (N) or hypoxia (H) for 48 h. Scale bars, 200 µm. (**d**) Quantification of invaded cells shown in (**c**). Mean ± SD of n = 3. NS, not significant; ***P < 0.005. (**e**) Western blot analysis of HIF-1α expression in cells after incubation with (+) or without (−) 10 µM CQ under normoxia (N) or hypoxia (H) for 12 h. Prior to incubation with primary antibodies, the blotted membrane was stained with Ponceau dye (Supplementary Fig. S3).

Because we observed that MKN45 cells are protected against the effects of hypoxia on mtROS and show hypoxia-induced colocalisation of lysosomes and mitochondria, we next assessed the effects of inhibiting lysosomal function in these cells by incubation with CQ, which inhibits lysosome acidification. CQ-treated MKN45 cells under hypoxia for 48 h showed increased mtROS production compared with untreated cells (Fig. 4a), whereas the untreated cells were unaffected by hypoxia (Fig. 4b). The same pattern of response to hypoxia and CQ treatment was observed in Transwell invasion assays (Fig. 4c,d), indicating that functioning lysosomes are necessary for MKN45 cells to prevent hypoxia-induced increases in mtROS and cell invasion. CQ treatment had no effects on either the proliferation or the survival of MKN45 cells under normoxia or hypoxia (Supplementary Fig. S2) or on hypoxia-induced expression of HIF-1α (Fig. 4e). Collectively, these findings suggest that the inhibition of lysosome function by CQ treatment elevates mtROS generation and promotes the invasiveness of hypoxic MKN45 cells.

### Assessment of hypoxia-induced mitophagy in GC cells

**Figure 5:**
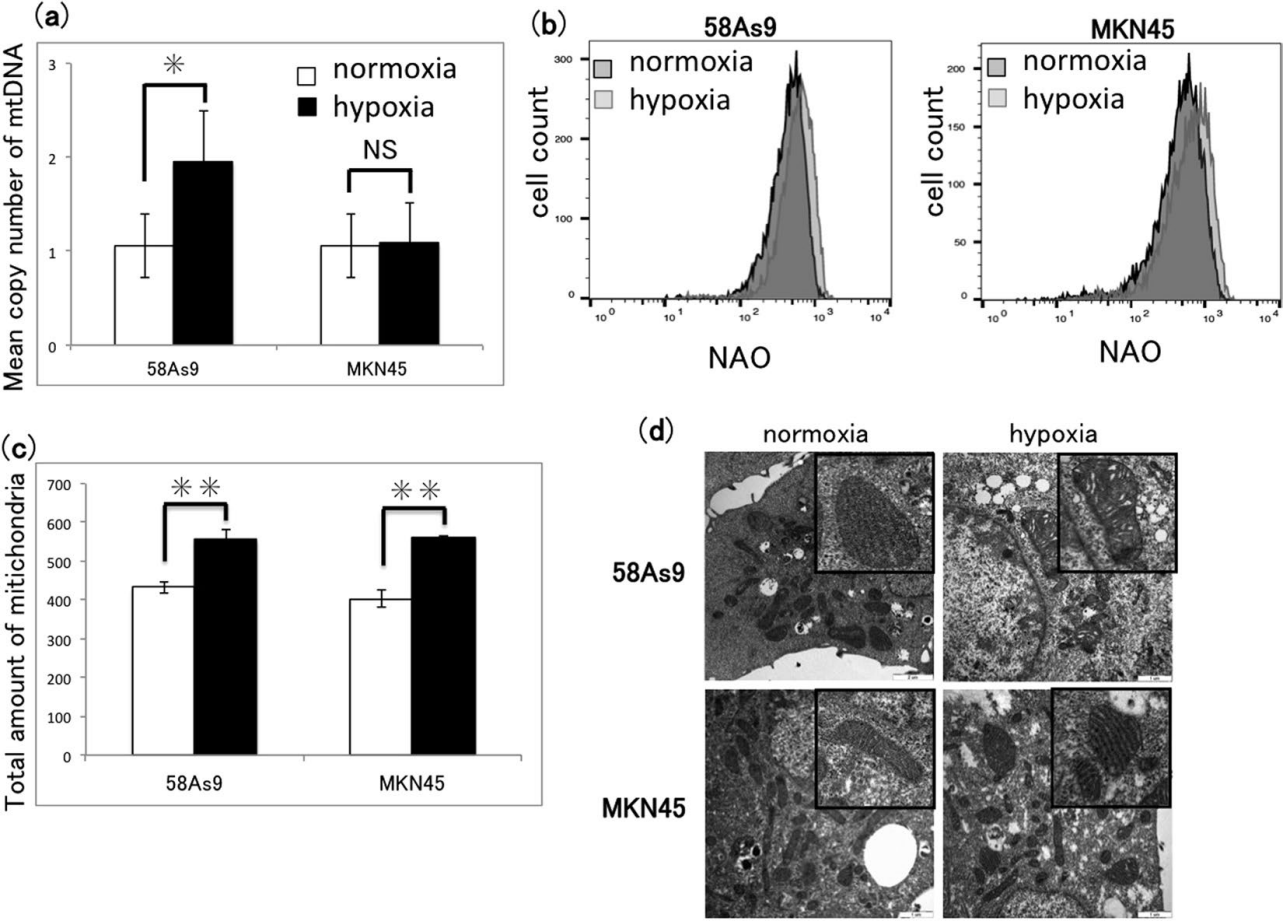
Analysis of mitophagy in GC cells under hypoxia or normoxia. (**a**) 58As9 and MKN45 GC cells were incubated under normoxia or hypoxia for 48 h. Mean copy number of mtDNA was estimated by quantitative PCR analysis and plotted in a graph. Mean ± SD of n = 6. NS, not significant; *P < 0.05. (**b**) Flow cytometric analysis of mitochondrial mass measured by nonyl acridine orange (NAO) staining of GC cells after incubation under normoxia or hypoxia for 48 h. (**c**) Quantification of mitochondrial mass shown in (**b**). Mean ± SD of n = 3. **P < 0.01. (**d**) Electron microscopic analysis of mitochondrial morphology in GC cells after incubation under normoxia or hypoxia for 48 h. Insets show magnifications of mitochondria. Scale bars, 1 µm.

We next determined whether mitophagy was activated in hypoxia-exposed 58As9 or MKN45 cells. Mitophagy eliminates damaged mitochondria via engulfment in autophagosomes, leading to reductions in mtDNA copy number and mitochondrial mass. The results showed that the mean copy number of mtDNA was significantly higher in 58As9 cells incubated for 48 h under hypoxia than for those under normoxia, whereas the copy number did not differ between normoxic and hypoxic MKN45 cells (Fig. 5a). We also measured the mitochondrial mass in GC cells by staining with nonyl acridine orange (NAO), a metachromatic fluorescent dye that binds to mitochondria regardless of their membrane potential. As shown by the flow cytometry profiles, mitochondrial mass was slightly elevated in both cell lines after 48 h of incubation under hypoxic compared with normoxic conditions, and there was no apparent difference between the cell lines (Fig. 5b). In both cell lines, the total amount of NAO was significantly increased under hypoxia, compared with that under normoxia (Fig. 5c). Next, we analysed hypoxia-induced changes in mitochondrial morphology by electron microscopy. In both cell lines cultured under normoxia, the mitochondrial cristae appeared as dense and regularly folded structures; however, cristae were sparser and more irregularly shaped in 58As9 cells under hypoxia for 48 h, whereas those in MKN45 cells were relatively unaffected (Fig. 5d). In addition, we did not observe mitochondrial engulfment by autophagosomes in either cell type under normoxia or hypoxia (Fig. 5d). Taken together, these findings indicate that mitophagy may not be induced by hypoxia in either GC cell line.

### Investigation of Mieap expression and hypoxia-induced MALM

**Figure 6:**
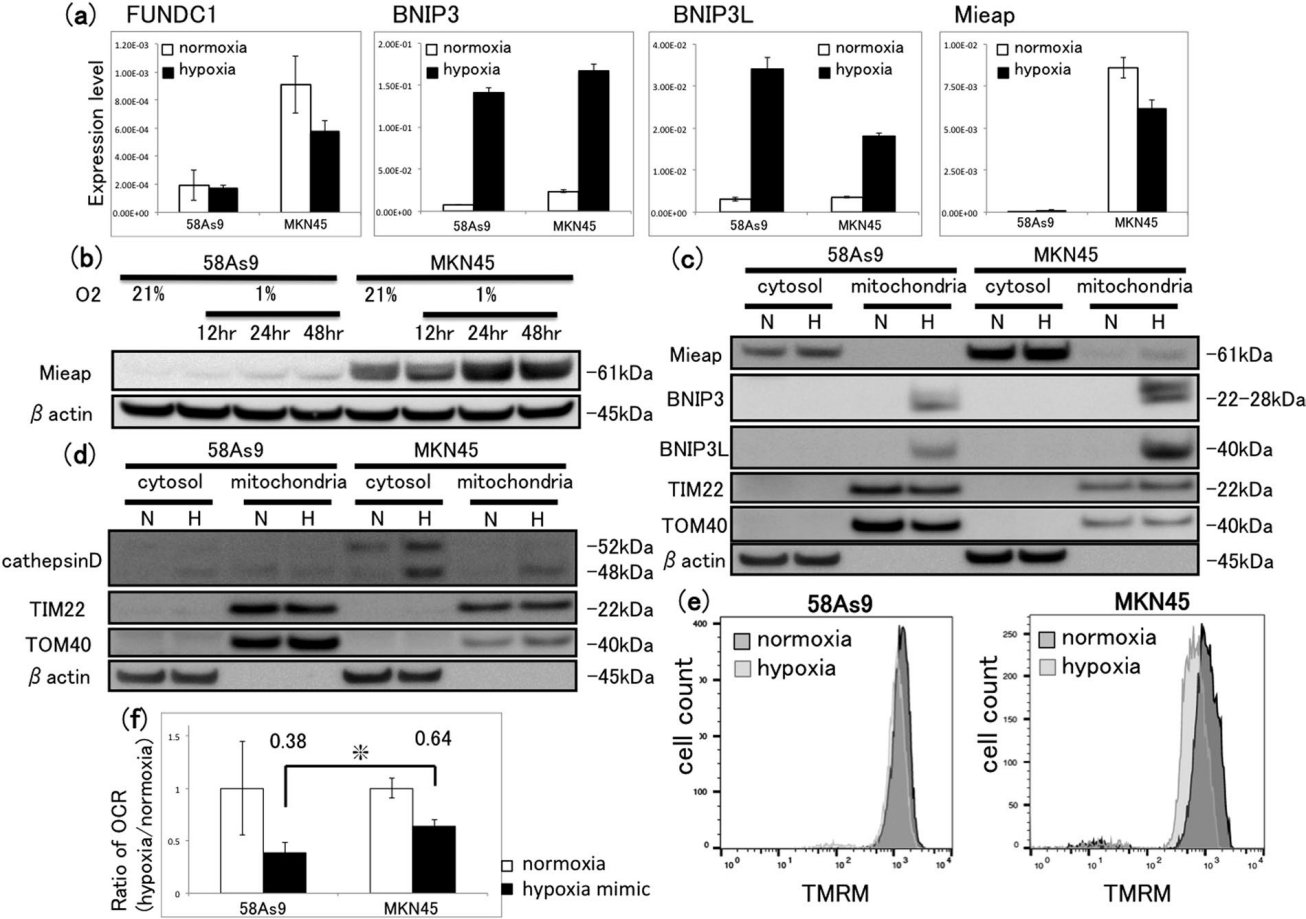
Assessment of molecular changes associated with mitophagy and MALM in GC cells. (**a**) RT-qPCR analysis of mitophagy- or MALM-related gene expression in 58As9 and MKN45 cells after incubation under normoxia or hypoxia for 24 h. Mean ± SD of n = 3. (**b**) Western blot analysis of Mieap in whole-cell lysates of GC cells after incubation under normoxia for 24 h or hypoxia for 12–48 h. Prior to incubation with primary antibodies, the blotted membrane was stained with Ponceau dye (Supplementary Fig. S4). (**c**) Western blot analysis of Mieap, BNIP3 and BNIP3L proteins in fractionated lysates of GC cells after incubation under normoxia or hypoxia for 48 h. TIM22 and TOM40 are markers for the mitochondrial fraction, and β-actin is a marker for the cytosolic fraction. A nonspecific signal that does not reflect Mieap is present in the cytosolic fraction of 58As9 cells. Prior to incubation with primary antibodies, the blotted membrane was stained with Ponceau dye (Supplementary Fig. S5). (**d**) Western blot analysis of cathepsin D in fractionated lysates of GC cells after incubation under normoxia or hypoxia for 48 h. Prior to incubation with primary antibodies, the blotted membrane was stained with Ponceau dye (Supplementary Fig. S6). (**e**) Flow cytometric analysis of the mitochondrial membrane potential integrity assessed by TMRM staining in GC cells after incubation under normoxia or hypoxia for 72 h. (**f**) Ratio of the oxygen consumption rate (OCR; µs/µg protein) by 58As9 and MKN45 cells after incubation under normoxic or hypoxia-mimicking (CoCl_2_) conditions for 24 h. Numbers above the bars indicate the mean OCR ratio (OCR under hypoxia/normoxia) for each cell line. Mean ± SD of n = 3. *P < 0.05.

We next analysed the expression of several MQC-related genes involved in mitophagy (FUNDC1, BNIP3 and BNIP3L) and MALM (Mieap). As shown in Fig. 6a, each of FUNDC1, BNIP3 and BNIP3L mRNA was expressed in both 58As9 and MKN45 cells, and the expression of BNIP3 and BNIP3L mRNA was strongly induced by hypoxia. In contrast, Mieap mRNA was detectable only in MKN45 cells, and there was no apparent difference between the levels detected under normoxia or hypoxia (Fig. 6a). Consistent with this, western blot analysis revealed that Mieap protein was expressed in MKN45 cells, but not in 58As9 cells, exposed to normoxia or hypoxia for 12–48 h (Fig. 6b). Moreover, analysis of fractionated cell lysates indicated that, although Mieap protein was predominantly cytosolic in MKN45 cells, hypoxia induced its appearance in the mitochondrial fraction of MKN45 cells, but not that of 58As9 cells (Fig. 6c). Consistent with the qRT-PCR analyses, expression of the HIF-1α target genes BNIP3 and BNIP3L was induced by hypoxia in the mitochondrial fractions of both GC cell lines (Fig. 6c). To assess the mechanism of MALM, we examined the subcellular localisation of lysosomes and the function of mitochondria in hypoxia-exposed cells in more detail. As measured by western blot analysis, expression of the lysosomal protein cathepsin D was not detected in the cytosolic or mitochondrial fractions of 58As9 cells incubated under normoxia or hypoxia for 24 h (Fig. 6d). In contrast, the protein was detectable in the cytosol but not the mitochondria of MKN45 cells under normoxia, but the expression was increased in both compartments after exposure to hypoxia (Fig. 6d), thus confirming the localisation of lysosomes to the mitochondria only in hypoxic MKN45 cells. To assess the effects of hypoxia on mitochondrial function, we measured the mitochondrial membrane potential (MMP) and O_2_ consumption rate (OCR) in both cell lines. MMP was estimated by staining cells with tetramethylrhodamine (TMRM), a dye that accumulates in mitochondria in an MMP-dependent manner. We found that exposure to hypoxia for 72 h induced a stronger decrease in the MMP in MKN45 cells than in 58As9 cells (Fig. 6e). OCR was measured using a fluorescent assay under normoxia or hypoxia-mimicking conditions (CoCl_2_), and the OCR ratio under hypoxia compared with normoxia was estimated. This analysis indicated that the OCR ratio was significantly higher in MKN45 than in 58As9 cells (Fig. 6f). Collectively, these findings suggest that MALM is induced in MKN45 cells under hypoxia, while the mechanism is impaired in 58As9 cells.

### Analysis of cell invasion and mtROS accumulation in Mieap-knockdown MKN45 cells

**Figure 7:**
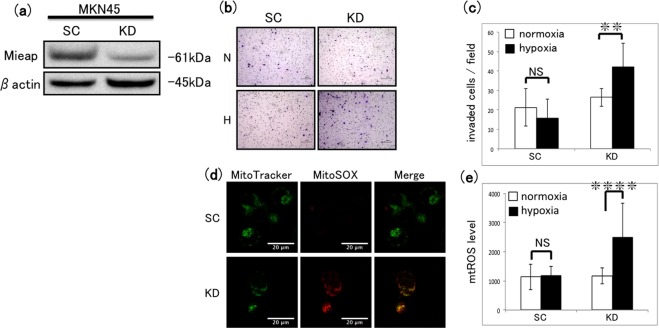
Effect of Mieap knockdown in MKN45 cells on cell invasion and mtROS generation. (**a**) Western blot analysis of Mieap expression in MKN45 cells stably expressing scrambled shRNA (SC) or Mieap-specific shRNA (KD) under normoxic conditions. Prior to incubation with primary antibodies, the blotted membrane was stained with Ponceau dye (Supplementary Fig. S7). (**b**) Transwell invasion assay of SC and Mieap KD MKN45 cells after incubation under normoxia (N) or hypoxia (H) for 48 h. Scale bars 200 µm. (**c**) Quantification of invaded cells shown in (**b**). Mean ± SD of n = 3. NS, not significant; **P < 0.01. (**d**) Fluorescence images of SC and KD cells after incubation under normoxia or hypoxia for 48 h and double staining with MitoTracker and MitoSOX-Red. Scale bars 20 µm. (**e**) Quantification of mtROS levels in SC and KD cells after incubation under normoxia or hypoxia for 48 h. Mean ± SD of n = 3. NS, not significant; ****P < 0.001.

Next, we determined the functional effects of disrupting MALM-mediated MQC by establishing MKN45 cells with the stable expression of Mieap-specific shRNA or scrambled shRNA (KD or SC cells, respectively). At this point, two clones (clones 1 and 2) were actually established by transfection using different sequences for Mieap-specific shRNA. Clone 2, exhibiting stronger inhibition of Mieap expression than clone 1, was designated as KD cells, and used in further study (Supplementary Fig. S9). Mieap expression was confirmed by western blot analysis to be strongly reduced in KD cells compared with that in SC cells (Fig. 7a). Intriguingly, Mieap KD significantly increased MKN45 cell invasion under hypoxia compared with that under normoxia, whereas invasion by control SC cells was unaffected by hypoxia (Fig. 7b,c). Consistent with this, mtROS levels were elevated by hypoxia in KD cells, but not in SC cells (Fig. 7d,e). These findings suggest that MALM requires Mieap expression and plays a pivotal role in preventing the hypoxia-induced increase in mtROS generation and cell invasion in MKN45 cells.

### Investigation of MALM activity in MKN45 cells with Mieap knockdown

**Figure 8:**
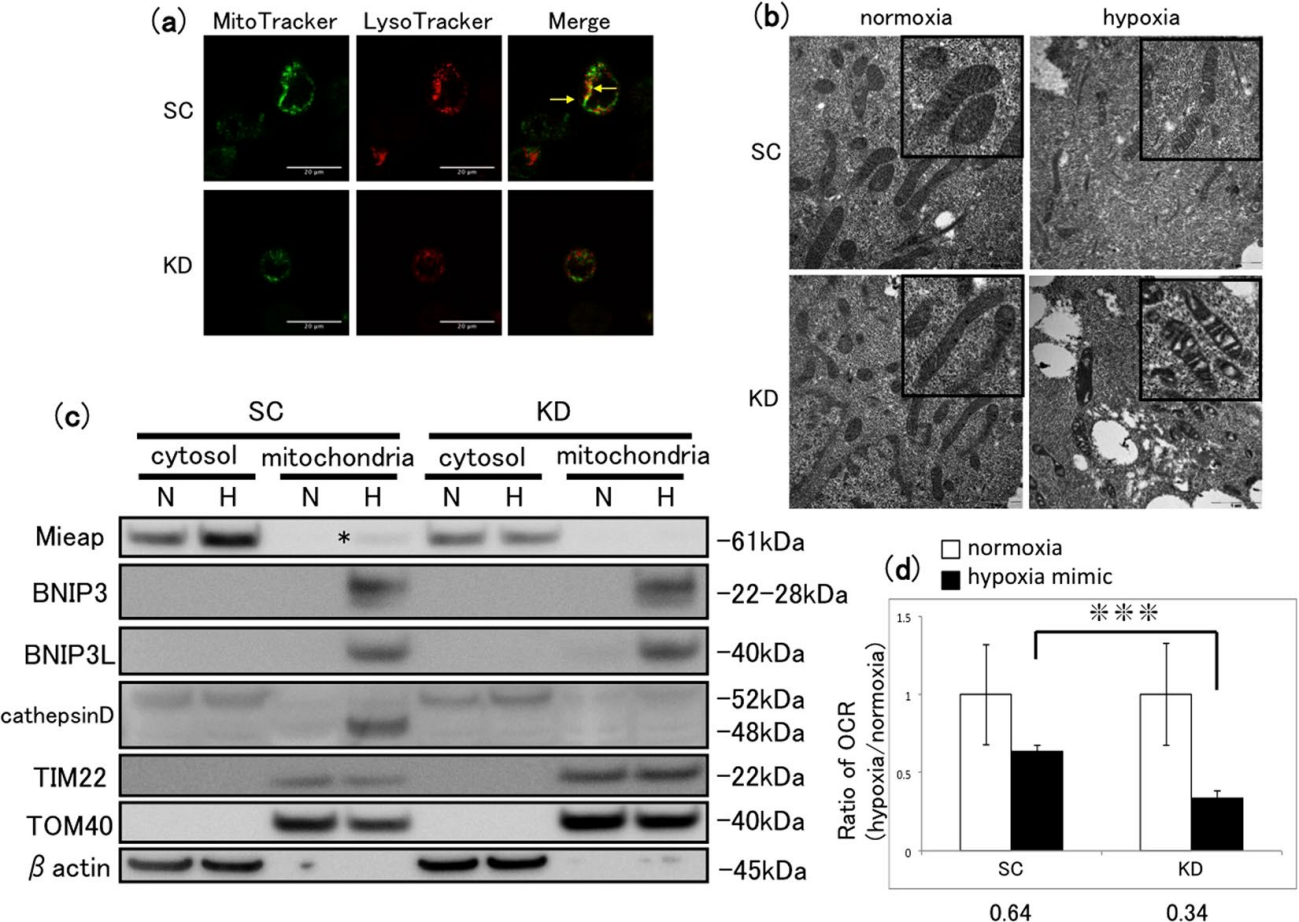
Assessment of MALM status in Mieap KD MKN45 cells. (**a**) Fluorescence images of SC or Mieap KD cells after incubation under hypoxia for 48 h and double staining with MitoTracker and LysoTracker. Arrows in the merged image indicate colocalised lysosomes and mitochondria. Scale bars, 20 µm. (**b**) Electron microscopic analysis of SC and Mieap KD cells after incubation under normoxia or hypoxia for 48 h. Insets show magnifications of mitochondria. Scale bars, 1 µm. (**c**) Western blot analysis of Mieap, BNIP3, BNIP3L and cathepsin D in fractionated lysates from SC and Mieap KD cells after incubation under normoxia or hypoxia for 24 h. Asterisk indicates a weak Mieap band in the mitochondrial fraction of hypoxic SC cells. Prior to incubation with primary antibodies, the blotted membrane was stained with Ponceau dye (Supplementary Fig. S8). (**d**) Ratio of OCR (µs/µg protein) of SC and Mieap KD cells after incubation under normoxic or hypoxia-mimicking (CoCl_2_) conditions for 24 h. Mean ± SD or n = 3. Numbers below the graph indicate the mean OCR ratios (OCR under hypoxia/normoxia) for each cell line. ***P < 0.005.

Finally, we analysed the effects of Mieap KD on MALM at the molecular level in MKN45 cells. Using the fluorescence MitoTracker/LysoTracker assay, we observed that hypoxia induced the colocalisation of lysosomes and mitochondria in hypoxic SC cells, while this was largely abolished in KD cells (Fig. 8a). Electron microscopy revealed that the morphological features of mitochondria in SC cells exposed to normoxia and hypoxia were similar; however, the mitochondrial cristae were sparser and more irregularly shaped under hypoxia than under normoxia in KD cells (Fig. 8b). Moreover, western blot analysis revealed weak Mieap expression in the mitochondrial fraction of SC cells, but not that of KD cells, under hypoxia (Fig. 8c); similarly, cathepsin D was expressed in the mitochondrial fraction only of SC cells under hypoxia (Fig. 8c). Lastly, we analysed the OCR in SC and KD cells under normoxic and hypoxia-mimicking conditions. As shown in Fig. 8d, the OCR ratio in KD cells was significantly lower than that in SC cells. In conclusion, these findings suggest that Mieap KD inhibits significant features of MALM in MKN45 cells under hypoxia.

## Discussion

Tumour hypoxia is known to induce mtROS accumulation and to promote cancer cell survival and progression^3,4^. The goal of this study was to elucidate the mechanism by which mtROS increase the invasive capacity of GC cells under hypoxic conditions. We confirmed that exposure to hypoxia induced mtROS accumulation and invasion in 58As9 cells, but not in the poorly invasive MKN45 cell line. During mitophagy and MALM, mitochondria are targeted to lysosomes^21,22,30^. We found that mitochondria colocalised with lysosomes only in hypoxic MKN45 cells, indicating that the MQC system may be impaired in 58As9 cells.

To assess whether mtROS generation directly regulates hypoxia-induced cancer invasion, we treated the cells with the ROS scavenger NAC. This approach revealed a critical role for mtROS in the HIF-1α expression, invasion and proliferation of 58As9 cells under hypoxia. Previous studies have shown that HIF-1α upregulates genes involved in cancer invasion under hypoxia^14–16^, and ROS generation is known to increase HIF-1α stability via PHD2 inactivation^3,4^. One interpretation of our data is that the scavenging of mtROS by NAC decreased HIF-1α stability, thereby suppressing the invasion and proliferation of 58As9 cells under hypoxia. We also demonstrated that the inhibition of lysosomal function by CQ increased mtROS generation and invasion by hypoxic MKN45 cells, but did not affect HIF-1α expression, cell proliferation or cell death. These results indicate that hypoxia-induced mtROS promote the invasiveness of MKN45 cells and that lysosomal function is required for hypoxia-induced MQC in these cells.

We attempted to elucidate the mechanism of hypoxia-induced MQC in GC cells by exploring various aspects of mitophagy and MALM. Mitophagy involves engulfment and elimination of damaged mitochondria via autophagosomes, leading to reductions in mtDNA copy number and mitochondrial mass^21,22^.

However, we found that neither cell line showed a decrease in mean copy number of mtDNA or mitochondrial mass under hypoxia compared with that under normoxia. Unexpectedly, in 58As9 cells, the copy number of mtDNA was significantly increased under hypoxia compared with that under normoxia. Replication or repair of mtDNA may be promoted by mtROS-mediated damage to DNA in hypoxic 58As9 cells. In addition, total mitochondrial mass was also increased by hypoxia in both cell lines, suggesting that mitochondrial neogenesis may be enhanced by hypoxia. However, the mechanisms by which the copy number of mtDNA in 58As9 and mitochondrial mass in both cell lines were increased under hypoxia are currently unclear. Moreover, morphological changes in mitochondrial cristae were found in hypoxic 58As9 cells, but not in MKN45 cells, suggesting that mtROS generation may have destroyed the structure of cristae in hypoxic 58As9 cells. Notably, autophagosomes engulfing mitochondria were not observed in either cell line by electron microscopy^21,22,28^. Taken together, these findings suggest that hypoxia may not induce mitophagy in MKN45 cells.

We examined the mechanism of hypoxia-induced MQC in more detail by analysing the expression of genes related to mitophagy and MALM. Interestingly, BNIP3 and BNIP3L mRNA was detected in 58As9 and MKN45 cells and was elevated in response to hypoxia in both cell lines, whereas Mieap was expressed only in MKN45 cells and was not significantly changed under conditions of hypoxia compared with normoxia. Previous work showed that Mieap forms a complex with BNIP3 or BNIP3L on the mitochondrial outer membrane in γ-irradiated colon cancer cell lines^30,31^. During MALM, the interaction between Mieap and BNIP3 or BNIP3L may open a pore between the outer and inner mitochondrial membranes, thus enabling lysosomes to enter the mitochondria^30,31^. During this process, MMP is reduced without cell death^31^. In the present study, we found that Mieap was strongly expressed in the cytosol of MKN45 cells under normoxia and hypoxia, but it was detected in the mitochondrial fraction only under hypoxia. In addition, BNIP3 and BNIP3L were detected in the mitochondrial fraction of both cell lines under hypoxia, but not normoxia. These results imply that hypoxia may induce the expression of BNIP3 and BNIP3L on the mitochondrial outer membrane, leading to the recruitment of Mieap. The following findings from this study in combination suggest that MALM was operating in hypoxic MKN45 cells, but not in 58As9 cells: (i) cathepsin D was observed in the mitochondrial fraction of hypoxic MKN45 cells, but not in 58As9 cells; (ii) hypoxia induced a stronger reduction in the MMP in MKN45 cells than in 58As9 cells; and (iii) MKN45 cells displayed a higher OCR ratio (hypoxia/normoxia) than did 58As9 cells. Furthermore, cathepsin D may be transported into the mitochondria through mitochondrial membrane pores formed by Mieap–BNIP3 or Mieap–BNIP3L complexes. Cathepsin D may then digest oxidised proteins in the mitochondria, thereby contributing to the restoration of mitochondrial respiration, as previously reported to occur during MALM^31^.

The effects of Mieap KD identified in this study confirmed the involvement of MALM in the MQC in MKN45 cells. Compared with those in control SC cells, KD of Mieap resulted in significant elevations of mtROS generation and cell invasion under hypoxia; induced morphological changes in the mitochondrial cristae under hypoxia; inhibited cathepsin D protein expression in the mitochondrial fraction under hypoxia; and significantly decreased the OCR ratio (hypoxia/normoxia). Taken together, these results indicate that Mieap is required for hypoxia-induced MALM in MKN45 cells, which may regulate mtROS generation, leading to the suppression of cell invasion. However, further investigation will be necessary to confirm that lysosomal proteins are transported into the mitochondria (e.g., by proteinase K digestion analysis)^31^, and that complexes are formed between Mieap and BNIP3 or BNIP3L (e.g., by coimmunoprecipitation)^31^. Notably, Mieap expression is reportedly reduced by the mutation of TP53 or methylation of its promoter in colon cancer cells and tissues^29,34^. We found that Mieap mRNA was expressed in five of nine GC cell lines tested (including 58As9 and MKN45; data not shown). Previous studies reported that wild-type p53 was expressed in MKN45, while the status of TP53 in 58As9 has not been investigated^35,36^. Therefore, we analysed p53 expression in MKN45 and 58As9 cells by western blotting (Supplementary Fig. S10). The results showed that both cells expressed p53 under normoxia and hypoxia (Supplementary Fig. S10). Interestingly, positive p53 signal less than 53kd was observed in 58As9, compared with the molecular weight of p53 in MKN45 (Supplementary Fig. S10). The p53 antibody used in this study recognizes the N-terminal region of this protein. Therefore, some frameshift mutation in TP53, whereby a C-terminus-truncated p53 isoform is produced, may occur in 58As9 cells, as previously reported in high-grade serous ovarian cancer^37^. The TP53 mutation may result in the loss of function of p53 in 58As9. Furthermore, treatment with the DNA methyltransferase inhibitor 5-aza-dC did not increase Mieap mRNA expression in 58As9 cells (Supplementary Fig. S11). Based on these findings, we hypothesize that TP53 mutation, but not DNA methylation of the Mieap gene promoter, may suppress Mieap expression in 58As9. However, further analysis of the TP53 mutation or Mieap promoter methylation may be necessary to clarify the mechanism of Mieap loss in 58As9 cells.

In conclusion, we report the first demonstration that MALM, and not mitophagy, is induced in GC cells under hypoxic conditions and demonstrate that hypoxia-induced MALM requires Mieap expression. Finally, this noncanonical MQC mechanism may also control mtROS production and suppress cell invasion in GC cells in hypoxic environments.

## Methods

### Cell culture and reagents

The GC cell line 58As9 was kindly provided by K. Yanagihara (National Cancer Center Hospital, Kashiwa, Japan). 58As9 was established from parental HSC-58 (scirrhous gastric carcinoma-derived cell line), and was reported to be associated with high rates of fatal cancerous peritonitis and ascites in a mouse orthotopic implantation model^38^. Another GC cell line, MKN45 (poorly differentiated adenocarcinoma), was purchased from the RIKEN Cell Bank (Tsukuba, Japan). The cell lines were grown in complete culture medium [RPMI-1640 medium (Sigma-Aldrich, St. Louis, MO, USA) supplemented with 10% fetal bovine serum (FBS; Biowest, Nuaillé, France) and 100 µg/ml kanamycin (Meiji, Tokyo, Japan)] at 37 °C in a humidified atmosphere. For the experiments, cells were cultured under normoxic (5% CO_2_ in air) and hypoxic conditions (1% O_2_, 5% CO_2_ in N_2_). Chloroquine diphosphate (CQ), N-acetyl-l-cysteine (NAC) and 5-aza-2-deoxycytidine (5-aza-dC) were purchased from Sigma-Aldrich and used at final concentrations of 20 mM, 10 and 5 µM, respectively.

### *In vitro* invasion assays

GC cells were resuspended in serum-free RPMI-1640 culture medium (1 × 10^5^ cells/200 µl) and seeded into the upper chambers of BioCoat Matrigel Invasion Chambers (354480; Corning) in 24-well plates. Aliquots of 500 µl of the supernatant from cultures of the MRC5 lung cancer cell line were placed in the bottom chambers. Plates were incubated for 48 h in normoxic or hypoxic conditions, and then noninvading cells on the upper side of the filter were gently removed with a cotton swab. The invaded cells on the lower side of the filter were fixed in 4% paraformaldehyde for 15 min and then stained with a 0.1% crystal violet solution for 15 min. Using a light microscope, cells in three random fields were visualised and enumerated with ImageJ software. All experiments were performed in triplicate.

### Knockdown of Mieap

pKLO.1-hU6 Pur plasmids encoding Mieap-specific shRNAs [TRCN0000141572 (clone 1) and TRCN0000142712 (clone 2)] or a control scrambled shRNA (SHC002) were purchased from Sigma-Aldrich. Cells were transfected with the plasmids using Lipofectamine 3000 (Thermo Fisher Scientific, Tokyo, Japan), in accordance with the manufacturer’s instructions. Cells stably expressing the Mieap shRNA or control shRNA (referred to as SC) were selected using puromycin.

### Western blot analysis

Whole-cell lysates were prepared by the resuspension of cells in lysis buffer [150 mM NaCl, 50 mM Tris-HCl, pH 7.5, 2 mM EDTA, 1% Triton X-100, 1% sodium deoxycholate, 2% SDS, 28 µM PMSF and a protease inhibitor cocktail mix (Roche, Mannheim, Germany)]. The lysates were sonicated for 30 s and the supernatants were then removed. For experiments analysing fractionated lysates, a Mitochondria/Cytosol Fractionation Kit (BioVision, Milpitas, CA, USA) was used, in accordance with the manufacturer’s instructions. Western blot analysis was performed as previously described^39^. In brief, aliquots containing 20 µg of protein (or 10 µg of cytosol/mitochondrial fractionated protein) were separated on 5–20% Bis-Tris gels (Intertechno, Tokyo, Japan) and transferred to Hybond-ECL membranes (GE Healthcare, Little Chalfont, UK). Membranes were blocked with 5% skim milk or 2% bovine serum albumin in Tris-buffered saline–0.01% Tween 20 for 30 min, and then incubated overnight at 4 °C with the following primary antibodies: anti-HIF-1α (1:1000, 610958; BD Biosciences), anti-Mieap (1:1000, HPA036854; Sigma-Aldrich), anti-TOM40 (1:100, sc365466; Santa Cruz Biotechnology), anti-TIM22 (1:1000, ab167423; Abcam), anti-cathepsin D (1:1000, 66534-1-Ig; Proteintech), anti-BNIP3 (1:1000, #13795; Cell Signaling Technology), anti-BNIP3L (1:1000, #12396; Cell Signaling Technology), anti-p53 (1:200, sc-126; Santa Cruz Biotechnology) and anti-β-actin (1:10,000, AC15; Sigma-Aldrich). Membranes were then washed and incubated with the corresponding secondary antibodies (SouthernBiotech, Birmingham, AL, USA) and the signal was developed using ECL Prime Western Blotting Detection Reagent (GE Healthcare). Images were acquired using a LAS-3000 Imaging System (Fujifilm, Tokyo, Japan).

### Quantification of intracellular ROS levels by flow cytometry

Intracellular ROS levels were evaluated using a 2′,7′-dichlorofluorescein diacetate-based Total ROS Detection Kit (ENZO Life Sciences, Farmingdale, NY, USA), in accordance with the manufacturer’s instructions. In brief, at the end of the experiment, cells (1 × 10^5^/sample) were washed and resuspended in ROS detection solution. Fluorescence was detected using a FACSCalibur flow cytometer (Becton-Dickinson, San Jose, CA, USA) using excitation and emission wavelengths of 488 and 545 nm, respectively. Data were analysed using FlowJo version 10.0 software (FlowJo, Ashland, OR, USA). All experiments were performed in triplicate. Data are displayed as the geometric mean fluorescence.

### Quantitative RT-PCR

Total RNA was extracted from cells using Isogen II (Nippon Gene, Osaka, Japan), and aliquots of 1 µg per sample were reverse-transcribed using a ReverTra Ace kit (Toyobo). RT-qPCR was performed using the CFX Connect Real-Time PCR Detection System (Bio-Rad, Hercules, CA, USA) with SsoAdvanced Universal SYBR Green Supermix (Bio-Rad), in accordance with the manufacturer’s protocol. The PCR program was performed using two StepAmp procedures, as described previously^35^. β-Actin mRNA served as an internal control. Primers for *FUNDC1, BNIP3, BNIP3L*, *MIEAP* and *ACTB* were as follows: *FUNDC1* sense, 5′-GTAATGGGTGGCGTTACTGG-3′ and antisense, 5′-GCTTTGTTCGCTCGTTTCTT-3′; *BNIP3* sense 5′-ACCCTCAGCATGAGGAACAC-3′ and antisense 5′-CAGCAAATGAGAGAGAGCAGCA-3′; *BNIP3L* sense 5′-GATGTGGAAATGCACACCAG-3′ and antisense 5′-TACCCAGTCCGCACTTTTCT-3′; *MIEAP* sense 5′-ATGATTGCAAATACCGCCGC-3′ and antisense 5′-CGACTTACAGATCGCACCGA-3′; and *ACTB* sense 5′-ACGCCTCTGGCCGTACCACT-3′ and antisense 5′-TAATGTCACGCACGATTCCC-3′.

### Confocal laser microscopy of mitochondrial markers

Cells (3 × 10^5^/sample) were seeded in 35-mm glass dishes and cultured in normoxic or hypoxic conditions with or without drugs as indicated. Cells were then stained with 100 nM MitoTracker Green FM (M7514; Invitrogen) to detect mitochondria; 5 µM MitoSOX Red (M36008; Invitrogen) to detect mtROS; or 50 nM LysoTrackerRed DND-99 (L7528; Invitrogen) to detect lysosomes. Images were acquired using an LSM-880 confocal laser microscope (Carl Zeiss). For each sample, the mean mtROS levels in three fields were calculated using ZEN software.

### Assessment of mitochondrial DNA copy number

At the end of the experiment, total DNA was extracted from cells using a DNeasy Blood & Tissue Kit (Qiagen, Hilden, Germany). The ratio of mitochondrial mtDNA to nuclear DNA (mtDNA:nuDNA) was determined by quantitative PCR (qPCR) using a Human Mitochondrial DNA (mtDNA) Monitoring Primer Set (TaKaRa, Kusatsu, Japan), in accordance with the manufacturer’s instructions. The kits included primers for *ND1* and *ND5* as the mtDNA targets and *SLCO2B1* and *SERPINA1* as the nuDNA targets. In brief, qPCR analysis was performed using the primer sets of *ND1, ND5, SLCO2B1* and *SERPINA1*, and the threshold cycle (Ct) values were determined. Next, ΔCt1 (Ct value of *SLCO2B1* − Ct value of *ND1*) and ΔCt2 (Ct value of *SERPINA1* − Ct value of *ND5*) were calculated. Finally, the mean copy number of mtDNA was determined by calculating the mean value of 2^ΔCt1^ and 2^ΔCt2^.

### Measurement of mitochondrial mass and mitochondrial membrane potential (MMP) by flow cytometry

Mitochondrial mass was measured by staining cells with 20 nM nonyl acridine orange (NAO) (Thermo Fisher Scientific) in phosphate-buffered saline (PBS)/5% FBS at 37 °C for 15 min. MMP was measured by staining cells with 250 nM tetramethylrhodamine methyl ester (TMRM; Thermo Fisher Scientific) at 37 °C for 30 min in complete medium. Both sets of cells were analysed using FACSCalibur with excitation and emission wavelengths of 488 and 575 nm, respectively.

### Electron microscopy

Cells (3 × 10^5^/sample) were seeded in 60-mm dishes and cultured in normoxic or hypoxic conditions for 48 h. The cells were fixed in 3% glutaraldehyde in PBS at 4 °C for 2 h, washed with PBS, post-fixed in 1% OsO_4_ buffered with PBS for 2 h, dehydrated in a graded series of ethanol and then embedded in Epon812. Ultrathin sections (90 nm) were placed on copper grids, double-stained with uranyl acetate and lead citrate, and then observed using a transmission electron microscope (JEM-1400Flash; JEOL, Akishima, Japan).

### Measurement of oxygen consumption rate (OCR) under hypoxia-mimicking conditions with CoCl_2_

OCR was evaluated using a MitoXpress Xtra Oxygen Consumption Assay kit (Luxel Biosciences, Cork, Ireland). In brief, 5 × 10^4^ cells were seeded in 96-well black plates and cultured under normoxia with or without cobalt(II) chloride hexahydrate (CoCl_2_) for 24 h. The medium was exchanged for fresh culture medium, MitoXpress Xtra reagent was added to each well and the wells were sealed with two drops of pre-warmed HS Mineral Oil. Fluorescent readings were started immediately using a fluorescence plate reader (CLARIOstar; BMG LABTECH). The OCR was estimated using a Dual-Read TR-F (Lifetime) kit, in accordance with the manufacturer’s instructions. OCR is expressed as µs/µg protein. Data are expressed as the ratio of OCR under hypoxia-mimicking and normoxic conditions.

### Statistical analysis

Data are expressed as the mean ± standard deviation (SD) of the indicated number of replicates. Group differences were analysed using Welch’s t-test, and P < 0.05 was regarded as significant.

## Supplementary information

supplemental information

## Data Availability

The datasets generated and/or analysed during the current study are available from the corresponding author on reasonable request.
