# Supplementary material for: Mieap-induced accumulation of lysosomes within mitochondria (MALM) regulates gastric cancer cell invasion under hypoxia by suppressing reactive oxygen species accumulation
Source: Sci Rep. 2019 Feb 26;9:2822. doi: 10.1038/s41598-019-39563-x (PMC6391448; doi:10.1038/s41598-019-39563-x)
Supplement: Supplementary file 1 — supplemental information [file 41598_2019_39563_MOESM1_ESM.pdf]

Title

Mieap-induced accumulation of lysosomes within mitochondria (MALM) regulates gastric cancer cell invasion under hypoxia by suppressing reactive oxygen species accumulation

Keiichiro Okuyama[1], Yoshihiko Kitajima[1,2,*], Noriyuki Egawa[1], Hiroshi Kitagawa[1], Kotaro Ito[1], Shinichi Aishima[3], Kazuyoshi Yanagihara[4], Tomokazu Tanaka[1], and Hirokazu Noshiro[1]

[1]Department of Surgery, Saga University Faculty of Medicine, 5-1-1 Nabeshima, Saga, 849-8501, Japan

[2]Department of Surgery, National Hospital Organization Higashisaga Hospital, 7324 Harakoga, Miyaki-cho, Miyaki-gun, Saga, 849-0101, Japan

[3]Department of Pathology, Saga University Faculty of Medicine, 5-1-1 Nabeshima, Saga, 849-8501, Japan

[4]Division of Translational Research, Exploratory Oncology Research & Clinical Trial Center, National Cancer Center, 6-5-1 Kashiwanoha, Kashiwa-shi, Chiba, 277-8577, Japan

*Correspondence to: Yoshihiko Kitajima MD, PhD, Department of Surgery, Saga University Faculty of Medicine, 5-1-1 Nabeshima, Saga 849-8501, Japan

E-mail: kitajiy@hosp.go.jp

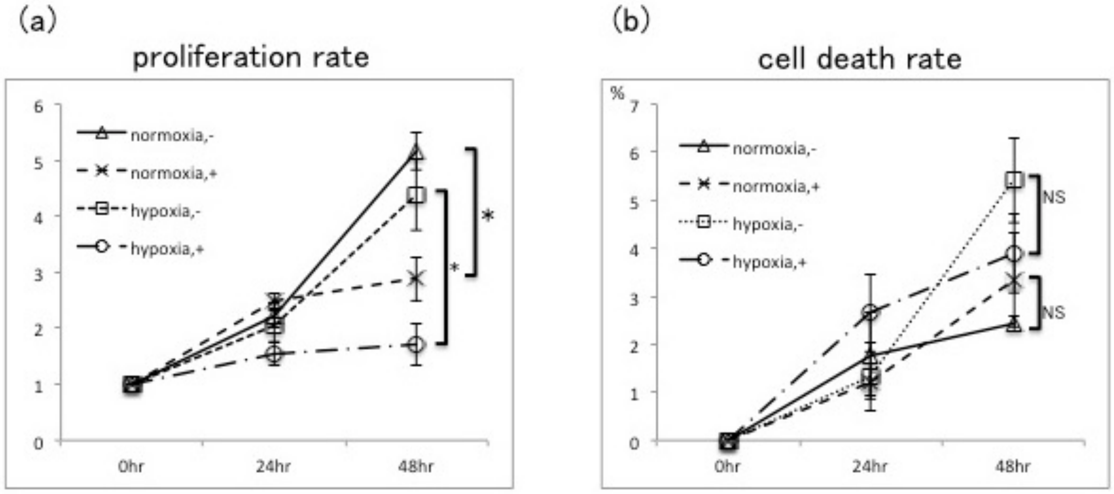

Figure S1. Effect of the ROS scavenger N-acetyl-L-cysteine (NAC) on the proliferation and survival of 58As9 GC cells under normoxia or hypoxia.

(**a and b**) 58As9 cells were incubated with (+) or without (−) 20 mM NAC under normoxia or hypoxia for 24 or 48 h. (a) Proliferation was measured by cell counting. (b) Cell viability was measured by Trypan blue staining. Results are expressed as the mean ± SD of triplicates relative to the 0 h time point. NS, not significant; *P<0.05.

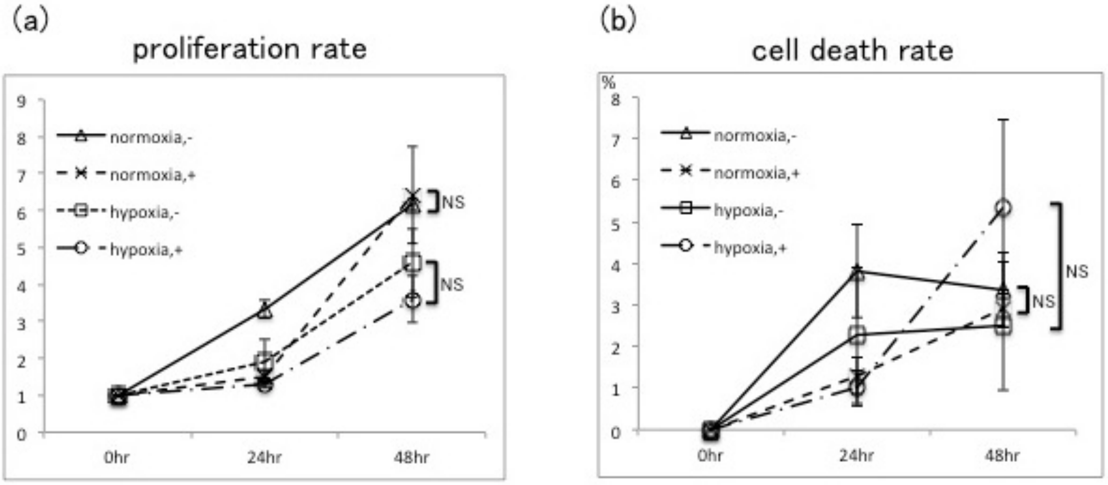

Figure S2. Effect of the lysosomal inhibitor chloroquine (CQ) on the proliferation and survival of MKN45 GC cells under normoxia or hypoxia. (**a and b**) MKN45 cells were incubated with (+) or without (−) 10 μμM CQ under normoxia or hypoxia for 24 or 48 h. (a) Proliferation was measured by cell counting. (b) Cell viability was measured by Trypan blue staining. Results are expressed as the mean ± SD of triplicates relative to the 0 h time point. NS, not significant.

Fig. 4 (e)

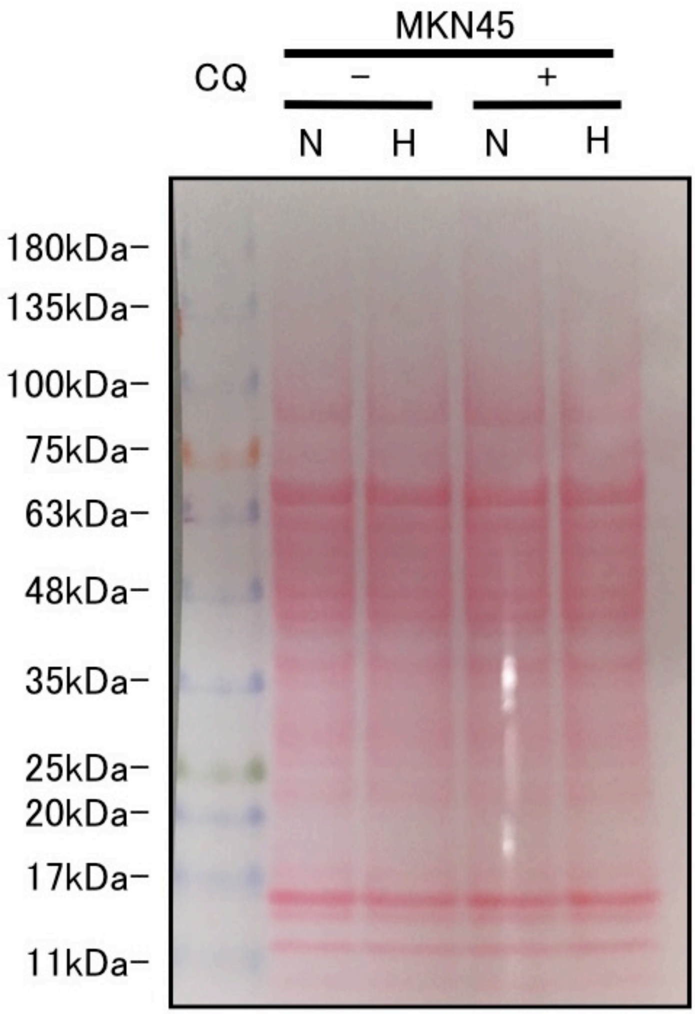

Figure S3. Protein staining with Ponceau dye blotted on Hybond-ECL membranes. Western blot analysis was entirely performed using the same membrane by cropping different parts.

.

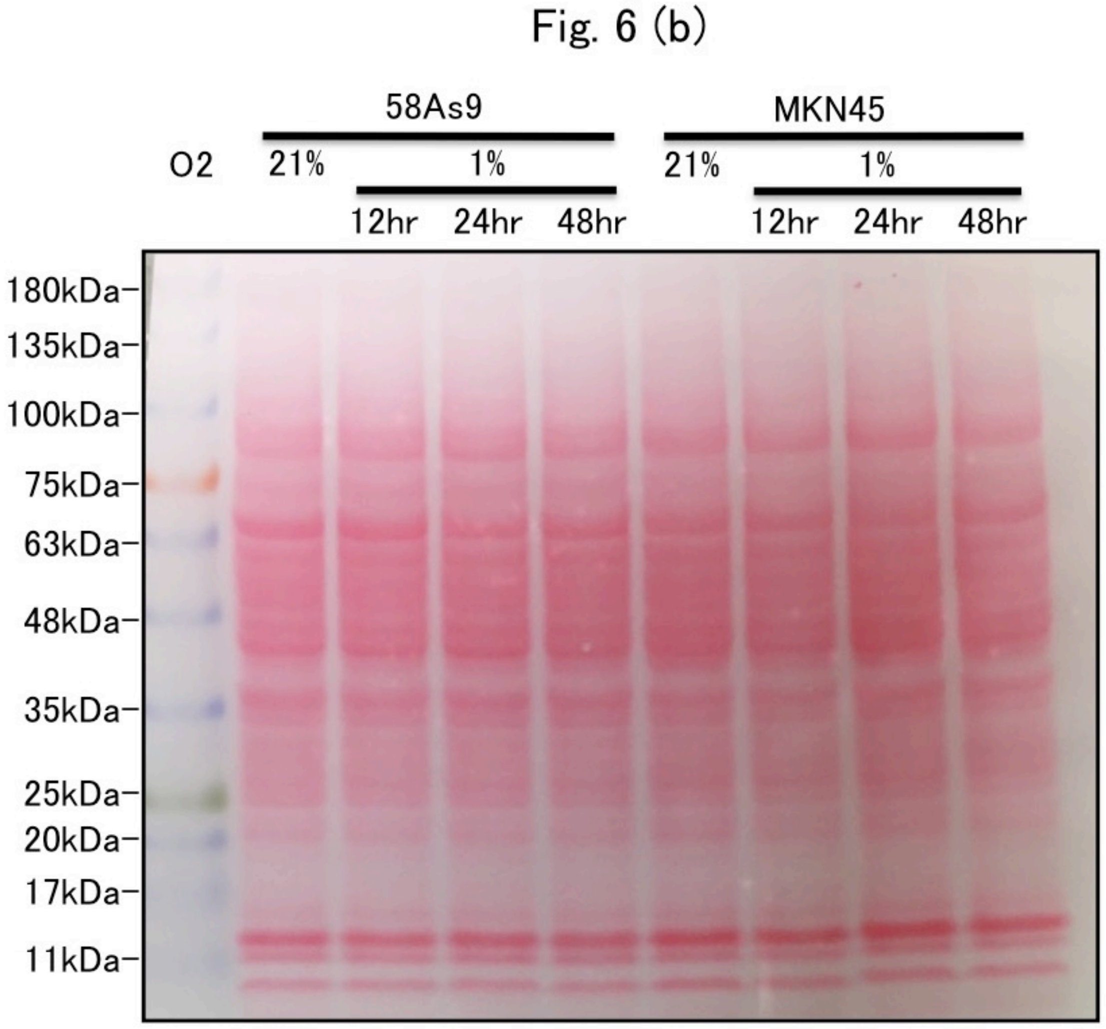

Figure S4. Protein staining with Ponceau dye blotted on Hybond-ECL membranes. Western blot analysis was entirely performed using the same membrane by cropping different parts.

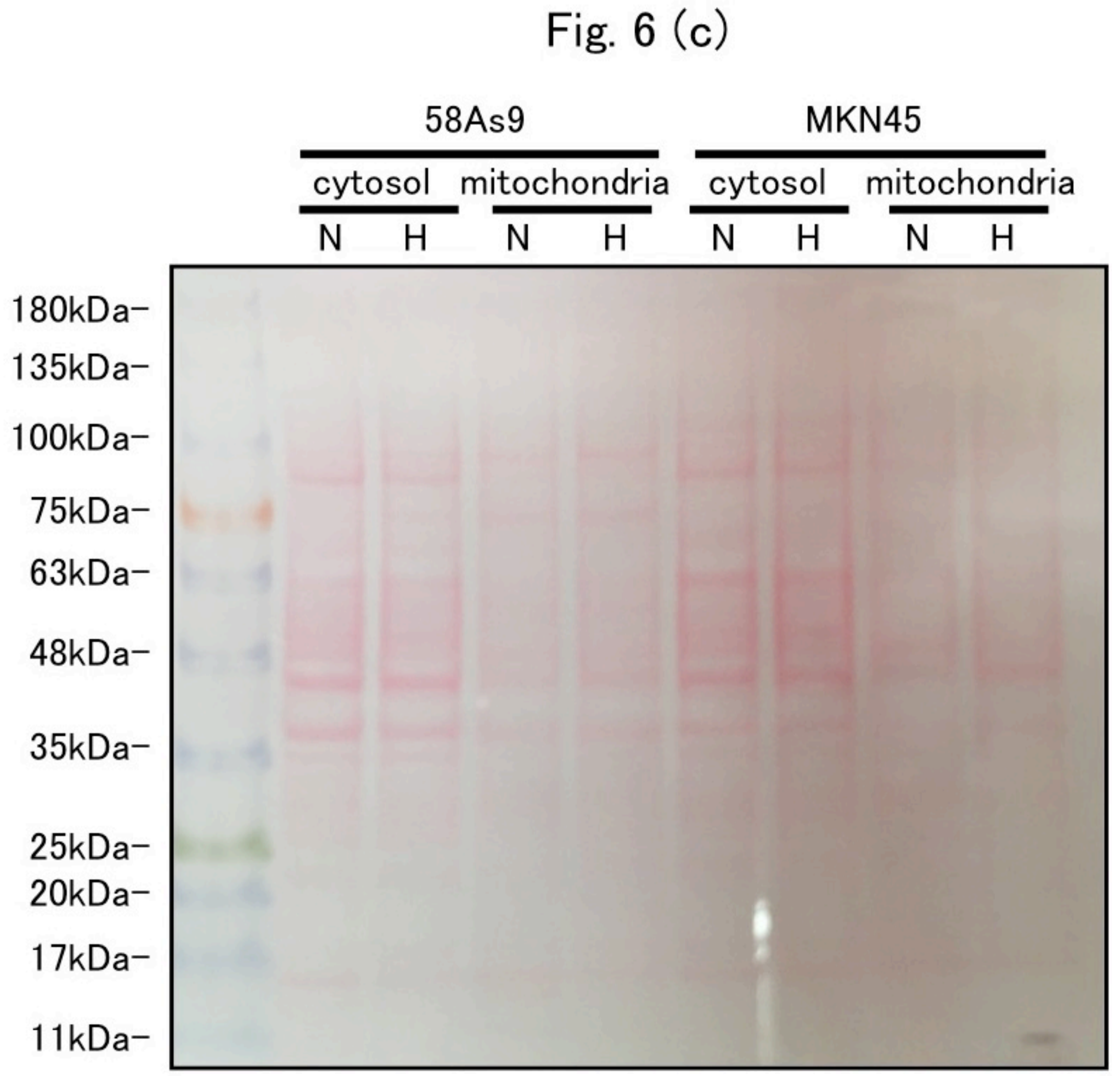

Figure S5. Protein staining with Ponceau dye blotted on Hybond-ECL membranes. Western blot analysis was entirely performed using the same membrane by cropping different parts.

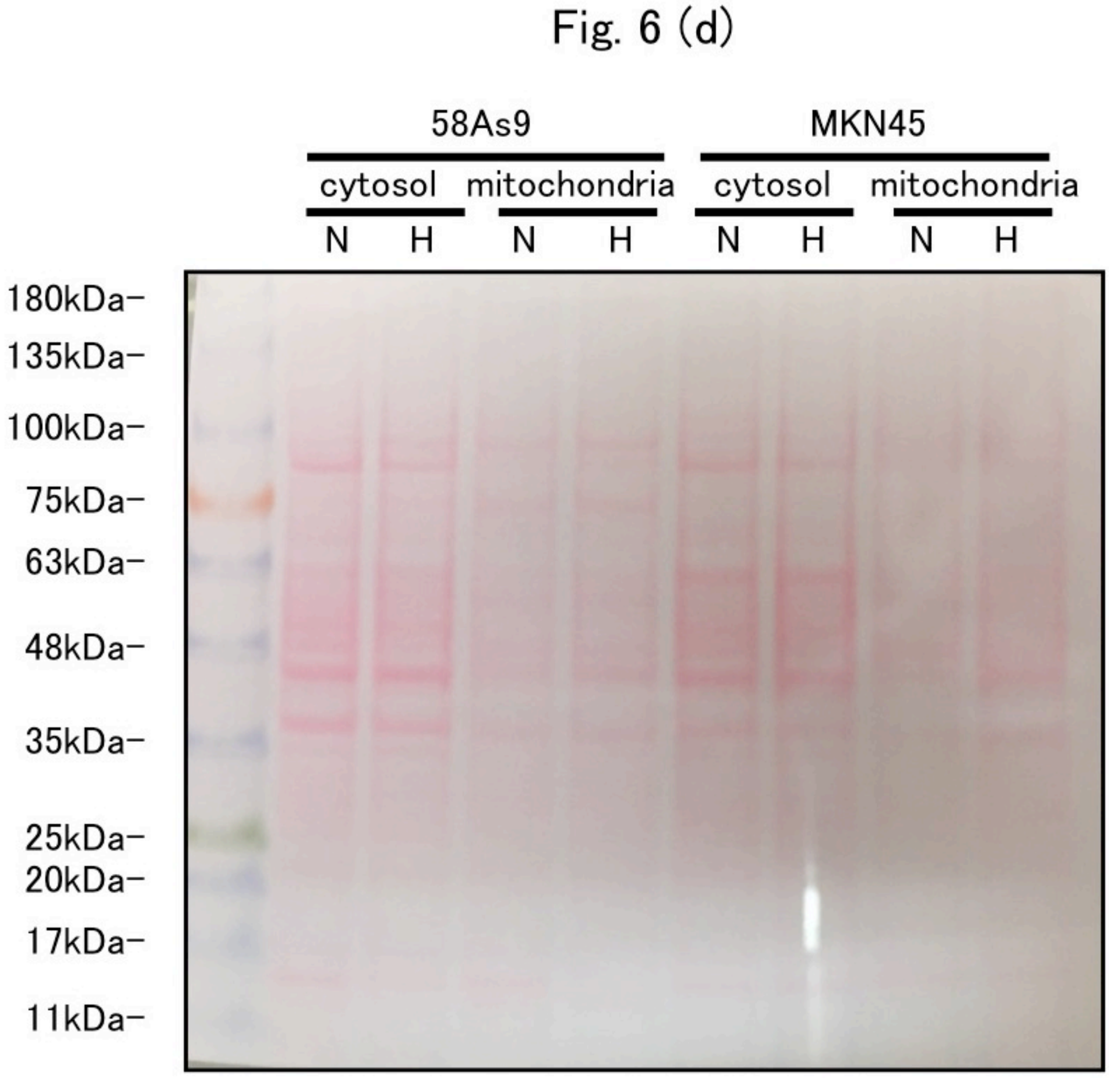

Figure S6. Protein staining with Ponceau dye blotted on Hybond-ECL membranes. Western blot analysis was entirely performed using the same membrane by cropping different parts.

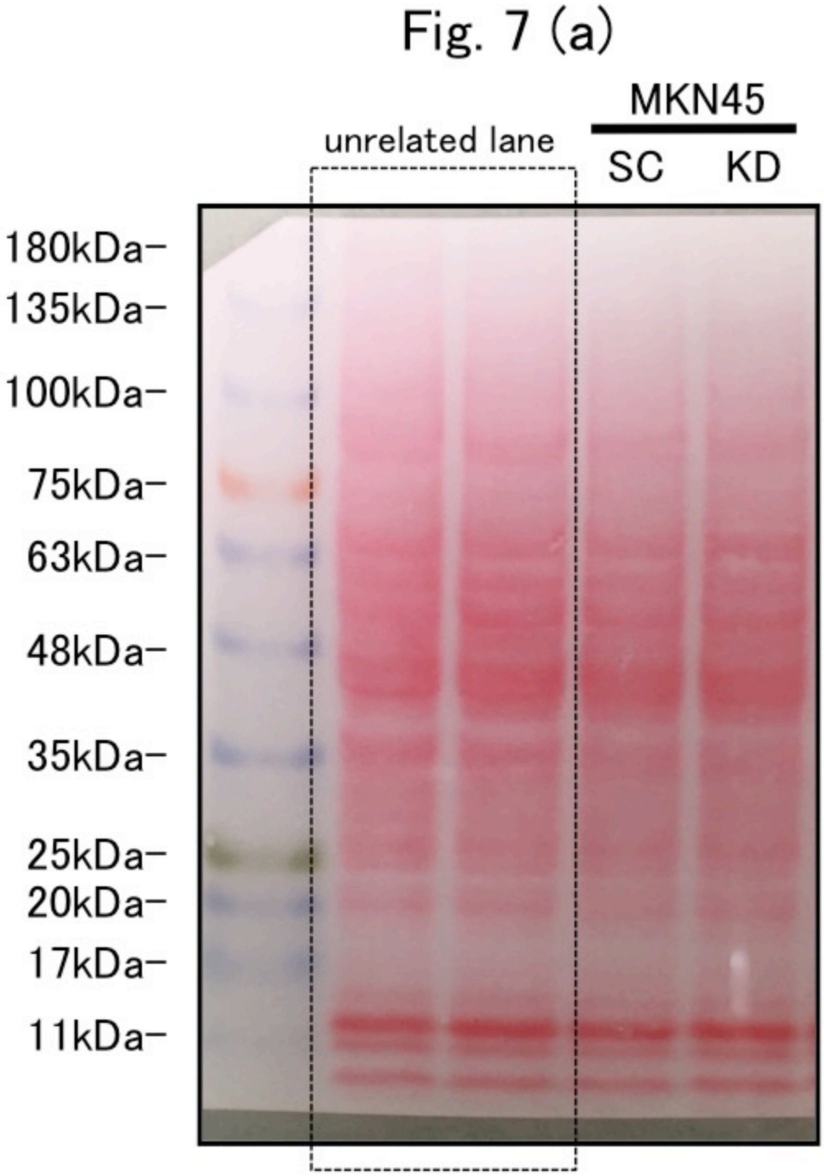

Figure S7. Protein staining with Ponceau dye blotted on Hybond-ECL membranes. Western blot analysis was entirely performed using the same membrane by cropping different parts.

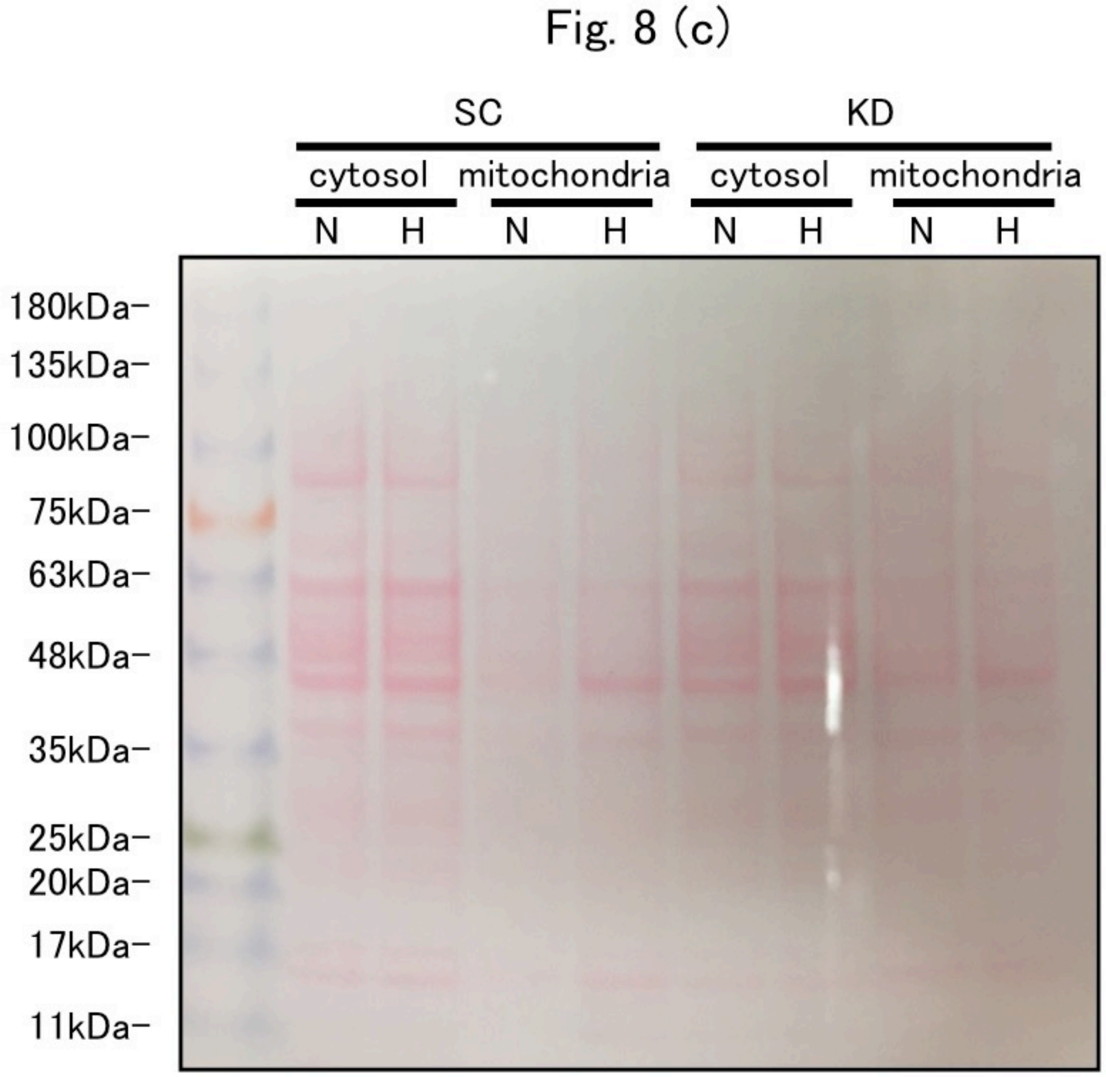

Figure S8. Protein staining with Ponceau dye blotted on Hybond-ECL membranes. Western blot analysis was entirely performed using the same membrane by cropping different parts.

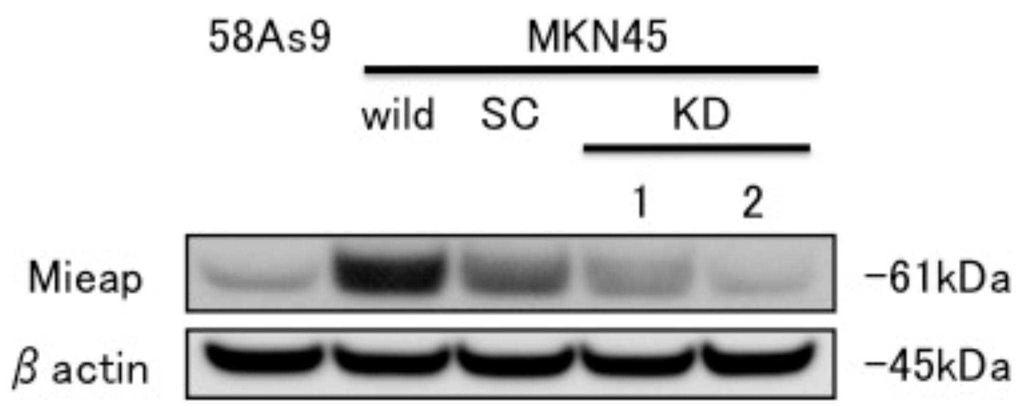

Figure S9. Western blot analysis of Mieap- knockdown clones in MKN45. Mieap- stable- knockdown clones (KD 1 and 2) were established. Clone 2 more strongly inhibited Mieap expression than clone 1.

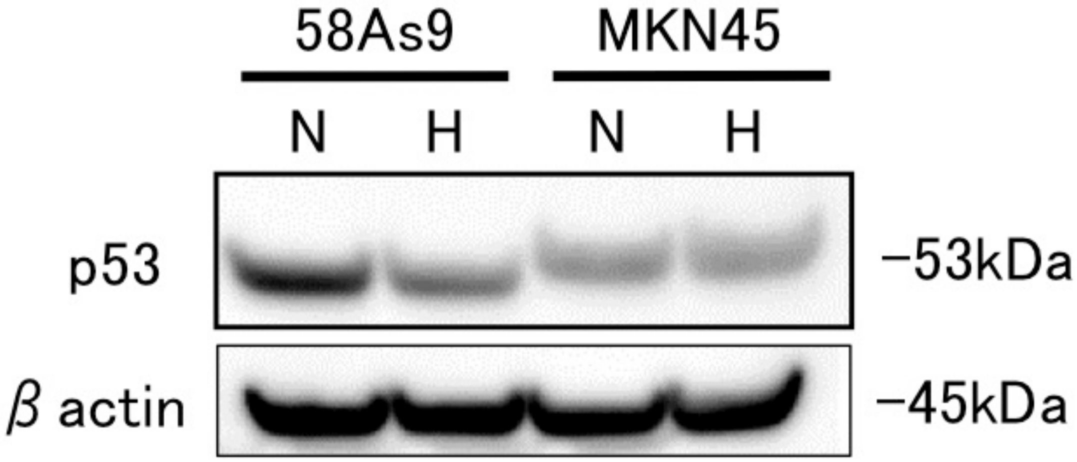

Figure S10. Western blot analysis of p53 in 58As9 and MKN45. The p53 expressions in 58As9 and MKN45 under normoxia (N) or hypoxia for 24 h (H) were is shown. Smaller size of p53 of less than 53 kDa was expressed in 58As9 cells.

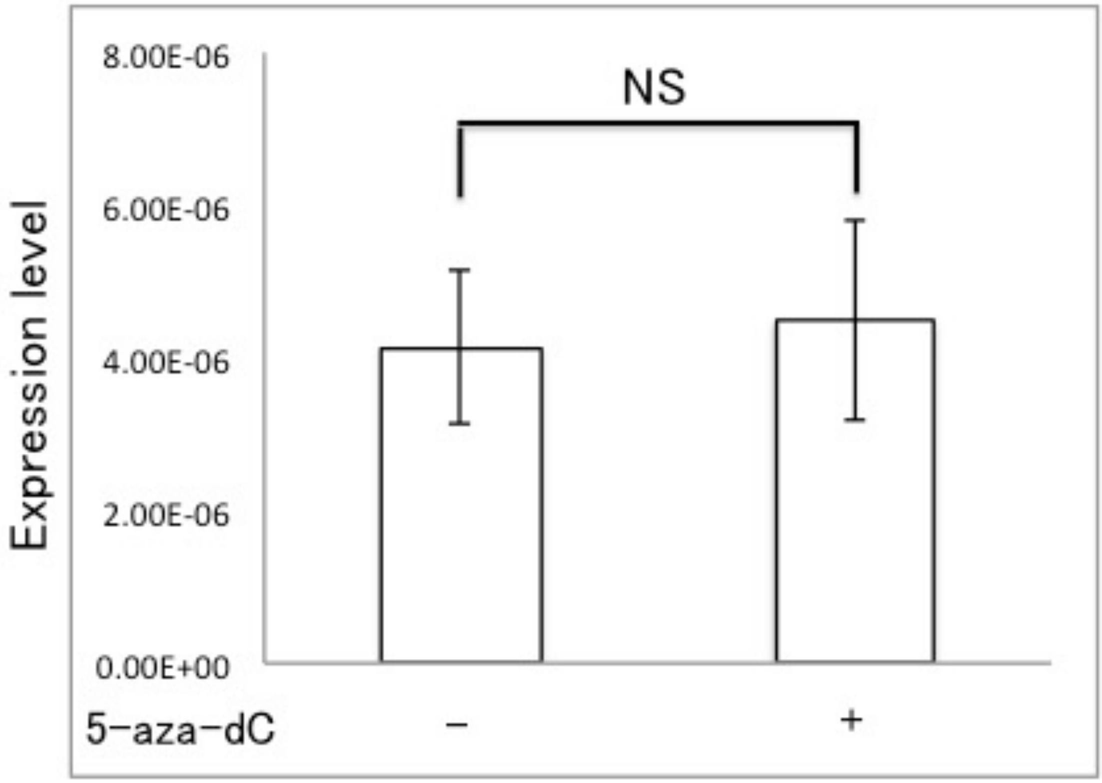

Figure S11. Expression levels of Mieap mRNA with or without 5-aza-dC in 58As9 in RT-qPCR. 58As9 were cultured with (+) or without (−) 5 μM of 5-aza-dC under normoxia for 24 h. Results are expressed as the mean ± SD in triplicates. NS, not significant.
